# Resistance and the delivery of healthcare in Australian immigration detention centres

**DOI:** 10.1007/s40592-023-00182-y

**Published:** 2023-10-09

**Authors:** Ryan Essex, Michael Dudley

**Affiliations:** 1 Institute for Lifecourse Development, University of Greenwich, Old Royal Naval College, Park RowLondon, London, SE10 9LS UK; 2 School of Psychiatry, University of New South Wales, High St, Kensington, NSW 2052 Australia

**Keywords:** Refugee, Asylum seeker, Health, Healthcare, Immigration detention, Resistance, Protest

## Abstract

There are few issues that have been as vexing for the Australian healthcare community as the Australian governments policy of mandatory, indefinite, immigration detention. While many concepts have been used to begin to describe the many dilemmas faced by healthcare professionals and their resolution, they are limited, perhaps most fundamentally by the fact that immigration detention is antithetical to health and wellbeing. Furthermore, and while most advice recognises that the abolition of detention is the only option in overcoming these issues, it provides little guidance on how action within detention could contribute to this. Drawing on the work of political theorists and the broader sociological literature, we will introduce and apply a form of action that has not yet been considered for healthcare workers within detention, resistance. We will draw on several examples from the literature to show how everyday resistance could be enacted in healthcare and immigration detention settings. We argue that the concept of resistance has several conceptual and practical advantages over much existing guidance for healthcare workers in these environments, namely that it politicises care and has synergies with other efforts aimed at the abolition of detention. We also offer some reflections on the justifiability of such action, arguing that it is largely consistent with the existing guidance produced by all major healthcare bodies in Australia.

## Healthcare in australian immigration detention

Australia has maintained a policy of mandatory immigration detention for three decades. The Australian government has overseen detention centres onshore, on mainland Australia and Christmas Island and offshore on Manus Island (Papua New Guinea) and Nauru. These policies have resulted in almost immeasurable human misery with riots, violence, abuse, self-harm and suicide all common (Dudley 2003). The express primary aim of these centres has been one of deterrence, something which has been made clear by successive governments. Unspoken operating principles also underwrite immigration detention and restrictive immigration policies, including neo-colonialism and racism. Lucrative detention contracts have tethered Australia’s ex-colonies (now sovereign low-income nations) Nauru and Papua New Guinea (Garton 2017). The socially pervasive malaise of racism is ubiquitous in immigration detention. Defined as negatively interpreting real or imagined ethnic group differences to legitimate discrimination and hostility (Ellefsen et al. 2022) racism manifests as disproportionate detention and spurious allegations of association with terrorism or crime against those from non-Western countries (Koutroulis 2009; Fiske 2012; Briskman 2020). These motivations, along with the devastating nature of these centres that has led a number of authors to draw parallels between Australia’s approach toward refugees and torture. Amnesty International (2016) concluded that, “refugees’ severe mental anguish, the intentional nature of the system, and the fact that the goal of offshore processing is to intimidate or coerce people to achieve a specific outcome – amounts to torture”. The International Criminal Court found that Australian immigration detention constituted ‘cruel, inhuman or degrading’ treatment (Doherty 2020). Australian Governments themselves fully understand and accept these policy-based injuries and sometimes deaths: they have reported and acknowledged the harms (Green and Eagar 2010; Australian Human Rights Commission 2014). Rather than act on these issues and take steps toward a more humane approach, the Australian government has instead dismissed such concerns and attacked critics, insisting that this approach is necessary as a means of deterring those who would otherwise seek Australia’s protection.

Australian healthcare professionals have been central to the day to day function of Australian immigration detention centres. Healthcare is provided on site with doctors, nurses, psychologists and counsellors employed. While clear cases of unethical behaviour have been documented, even those with the best intentions have found themselves limited in the care they can provide. Much has been written about healthcare within Australian immigration detention centres, and few contemporary issues have been as vexing for the Australian healthcare community. At the heart of these issues remains the fact that immigration detention is antithetical to health and wellbeing; it violates almost every human rights instrument to which Australia is signatory and is an affront to the dignity of those who are detained. Regardless of the actions taken by healthcare professionals working in the system, they can do little to avoid these issues, interventions are largely futile and their involvement is key to allowing the system to continue to function as it does. While much could be said about the actions taken by the healthcare community outside of detention that has sought to challenge and undermine these policies, like whistleblowing, marches and even civil disobedience (Essex 2020) the below discussion will be limited to the debates and strategies proposed for health workers within centres.

The conflict faced by health workers delivering health services in immigration detention has been conceptualised in a number of ways, as has the guidance proposed to mediate these conflicts. Arguably, the concept of dual loyalty has been most frequently utilised (Zion et al. 2012). Dual loyalty describes a situation where a health worker is faced with diverging or conflicting obligations between that of their patient and a third party, in the case of immigration detention, this is most commonly the Australian government or detention security contractors. While much has been said about the resolution of dual loyalty conflicts, the literature and guidance from professional bodies generally advises to place the interests of the patient first (Physicians for Human Rights 2002). While this might be helpful in other circumstances, such guidance provides little practical support for those working within detention centres, with healthcare professionals often unable to put their patients interests first because of the larger structural constraints placed upon them by working within detention (Martin 2018). Writing specifically about Australian immigration detention Briskman and Zion (2014) offer more pragmatic advice as to how clinicians could respond to the conflicts. They identify four possible responses which range from the delivery of care without any challenge to authorities, behaving in a mildly subversive manner to taking a more activist stance. They also identify ‘retreat’ as a possible response, that is, health workers may also decide to relinquish their role working in detention. They go on to argue that health workers should engage in both subversive and more openly activist actions while in detention, however do not elaborate on what this could look like in this context. The concept of complicity has also been used to a lesser extent to describe these conflicts (Essex 2016b; Jansen et al. 2018), in particular Lepora and Goodin’s (2013) framework of moral complicity. This framework provides a nuanced account of how to assess complicity with wrongdoing, identifying the factors that may increase complicity, such as knowledge of the wrongdoing and the contribution played in enabling the wrongdoing. It follows that after identifying the contribution made to wrongdoing, health workers could then take steps to minimise their complicity. While this framework has proven useful in examining questions about whether healthcare workers should continue to work in detention at all, for those who continue to do so minimising complicity with wrongdoing may not necessarily lead to better health or healthcare for those detained. Furthermore, (and similar to the issues present in resolving dual loyalty conflicts) the realities found within detention and restrictions placed on clinicians may mean that minimising complicity in all circumstances is not possible, and accentuates exposure to moral injury – the existential and spiritual distress arising from situational participation or exposure that transgresses personal values (Litz et al. 2009). Guidance elsewhere, from professional bodies like the Australian Medical Association, while covering a far more diverse range of issues, adds little to the discussions already in the literature. Like the advice related to dual loyalty almost every major Australian healthcare body calls for healthcare to be delivered to a standard equivalent to that found in the broader Australian community (Essex 2016a) and where conflicts are encountered, like the advice above, to put the interests of patients first (Essex 2019). A final form of action that has been discussed as a means to respond to Australian immigration detention has been a strike or boycott, that is, healthcare workers refusing to work in immigration detention centres until some type of minimum standard of care is met (Sanggaran 2016). While ongoing discussion about strike action should continue, such action appears unlikely in the foreseeable future (Essex 2018).

It is important to note that every author who has written or thought about these issues has recognised the importance of abolition and namely that these issues are likely to persist without broader structural change. Briskman and Zion (2014) for example suggest that “[f]or the ethical health worker, a focus on maintaining and incrementally improving the system is vexed and the aspiration must be the abolition of the detention system”. Even if abolition is our broader aim, in the absence of a strike and given the fact that health workers are likely to continue to work in immigration detention into the foreseeable future, the question remains, what can be done to facilitate the best care for detainees and ensure health workers are behaving as ethically as possible? We contend that much of the above guidance cannot be used in any meaningful way by those working within detention, furthermore it fails to take into account the day to day realities of working in detention.

Below we will introduce a form of action that has not yet been considered for health workers within immigration detention, everyday resistance. We will first discuss what everyday resistance is, how it has been conceptualised in the literature and some of its controversies. We will then draw on several examples from the literature to show how everyday resistance could be enacted in healthcare and immigration detention settings. We will also refer to the analogous concepts of everyday racism and everyday anti-racism, including as they apply in immigration detention. We will then offer some reflections on the advantages of engaging this concept, over some of the other guidance that already exists. While it will not remedy all of the issues we have discussed above, it has certain advantages over other guidance discussed above, namely that it politicises care and has synergies with other efforts aimed at abolition. Finally, we also offer some reflections on the justifiability of such action, arguing that it is consistent with the existing guidance produced by all major healthcare bodies.

## What is everyday resistance?

When we think of resistance, we might begin to think of public organised acts; protests, marches, sit-ins and even civil disobedience. In opposing Australian immigration detention, such resistance has been common in demanding reform. Within detention centres, while riots and protest have occurred, such action comes with risk and is often shut down quickly. The testimony from numerous healthcare professionals suggests even small acts of advocacy are often shut down (Martin 2018), and numerous staff have been stood down for what has been perceived to be advocacy (Koziol 2018; Doherty and Davidson 2016). In short, opportunities to openly oppose or undermine the system from within are limited and often come with high costs. Fortunately, public, visible and organised acts are only one type of resistance, resistance can occur day to day, in non confrontational forms, out of view of those in power.

While broader concept of resistance has been described as a “phenomenon with many faces” (Baaz et al. 2016) and as having a “palpable lack of definitional consensus” (Hayward and Schuilenburg 2014) and while many controversies remain about this concept in itself, this is not to say we cannot begin to outline its contours. The broader concept of resistance has been defined, specifically in relation to healthcare professionals as, “any act, performed by any individual (or collective) acting as or explicitly identifying as a healthcare professional, that is a response to power, most often in opposition to contentious, harmful or unjust rules, practices, policies or structures” (Essex 2021). This definition is obviously quite broad however, it encompasses but doesn’t adequately describe what is meant by everyday resistance.

Everyday resistance is one form of resistance, a form that has been underutilised and understudied as it relates to health and healthcare. The concept was introduced by Scott (1986) who identified it as a far more common response to oppression than open, public and collective acts of resistance. Everyday resistance can be contrasted to open, organised resistance, such as marches or civil disobedience. Everyday resistance is less visible than open, organised resistance, and often employed by groups who have relatively little power and thus luxury to openly confront their oppressors. Examples of actions include “foot-dragging, dissimulation, false-compliance, pilfering, feigned ignorance, slander, arson, sabotage, and so forth” (Scott 1986). While distinct, everyday resistance is not disconnected from more open, overt forms of resistance, as noted by Scott (1990) “each realm of open resistance to domination is shadowed by an infrapolitical twin sister who aims at the same strategic goals but whose low profile is better adapted to resisting an opponent who could probably win any open confrontation”.

Everyday resistance intersects major socially constructed identities– including subaltern, racial, ethnic, feminist, cultural, queer and others - and encompasses many areas of scholarship (Vinthagen and Johansson 2013). One field of everyday resistance which is particularly salient to restrictive immigration policies and immigration detention is everyday racism and anti-racism. First described by Essed (1991), ‘everyday racism’ designates verbal, behavioural and environmental racialized indignities that are brief, commonplace, subtle or apparently minor, and though often recurrent, sometimes go unremarked. As the counterpart of exceptional racism’s racist attacks, and the ‘macro’ of systematic or structural racism, the ‘micro’ of everyday racism involves daily ‘micro-aggressions’ that permeate society and its organisations, disadvantaging ethnic minorities (Reeders and Nguyen 2014) and causing harms. Minority group members experiencing everyday racism have offered a range of creative responses (e.g. ignoring, confronting, sharing experiences about, reporting and protesting (Ellefsen et al. 2022). Manifestations by health professionals resisting immigration detention are discussed below.

Since Scott’s introduction, the concept of everyday resistance has received considerable attention, with several points of contention for which debate continues to this day. While it is beyond the scope of this article to delve too deeply into the conceptual controversies related to resistance. it is worth touching on a few. The first issue related to such forms of resistance relates to visibility, that is, such forms of resistance are hidden and are not recognised by those in power. In addressing this point, Scott argues that it is problematic to assume that the most agreed upon, and therefore arguably the most “legitimate” forms of resistance, can be carried out by everyone. More often than not, the form of resistance depends on the form of power. That is, power (including the type of regime and the stakes involved, such as explicit threat to life) may constrain resistance; and many of the most oppressed do not have the luxury of organising public actions or engaging in civil disobedience for example. Scott goes on to note, if we are only concerned with organised public acts of resistance all we may be measuring is “the level of repression that structures the available options” (Scott 1989). A second major controversy again relates to recognition, but the recognition of the actors engaged in resistance, more specifically, do those resisting need to be doing so intentionally. This point has generated substantial discussion. On one side, without intent, resistance risks becoming vacuous with any number of actions potentially counting as resistance. On the other however, requiring intent privileges a certain type of resistance, that is, we risk missing a range of activities that undermine or oppose power. Baaz et al. (2016) argues that while knowing the intent of actors would be helpful in explaining resistance, intent should not be necessary for an act to be considered resistance. Furthermore, intent is difficult to predict or determine, even when we look to our own motives. As noted by Baaz et al. (2016), intent is “plural, complex, contradictory, or evolving as well as occasionally something that the actor is not sure about, views differently in retrospect, or even is not able to explain”. Ferrell (2019) argues that for most of us, most of the time, intent is too much to ask, but particularly for those who are most oppressed, noting that, “[i]f the requirement is that people must clearly verbalize their intent in order to be counted as resisters, this would seem to privilege those educated in the ways of discourse and debate and to disadvantage those for whom actions may indeed speak louder than words”. He concludes “ the whole standard of intentionality strikes me as elitist, intellectualist, and rationalist—a standard that perhaps tells us more about the scholars who require it of resistance that it does about those who engage in resistance directly”. A final point worth touching upon relates to the concept of everyday resistance itself and its relationship to power. A number of authors have more recently questioned the wisdom as conceptualising resistance as a binary between everyday acts and more public forms of resistance. Needless to say, several other conceptualisations now exist (Lilja 2022). In saying this however, there is generally agreement about many of the fundamental elements of everyday resistance, namely that resistance may not necessarily confront power and that it may be hidden.

## Everyday resistance in immigration detention

How might everyday acts of resistance manifest in Australian immigration detention centres? And how could health workers use this as a strategy in the delivery of care? Below we will provide some examples to begin to outline how resistance within detention may manifest. On this point it is worth starting with the broader literature that has explored everyday resistance in healthcare more generally. While this literature is limited, it does begin to provide an idea of what this action could be in a healthcare setting. A particularly interesting body of work has explored how Swedish General Practitioners (GPs) resisted the government’s austerity-charged gatekeeping of access to sickness benefits. In several papers Shutzberg (2021) details the strategies employed by doctors to ensure their certificates are accepted and that their patients receive benefits. While as a whole we might label such acts as subversion, the actions employed by GPs were far more nuanced, with at least eight strategies deployed; these included exaggerating patient symptoms, omission of certain details and utilising buzzwords that the government wanted to see on certificates (Shutzberg 2019). We can find examples elsewhere. Mainey et al. (2022) explored the provision of abortion care amongst nurses in rural Australia. While several barriers to providing care were found, more interesting were the strategies employed by nurses to ensure care was provided. These included subverting systems that restricted care, working around colleagues who were opposed to abortion and even networking with others outside the health service to ensure patients could travel for the care they sought. A final example comes from Shaw et al. (2018) who examined everyday resistance amongst medical students in the UK and Australia, particularly in relation to professionalism lapses of more senior medical staff. This study suggests that resistance is a frequent occurrence; medical students resisted verbally, directly and indirectly, subtly through presenting concerns or making suggestions, through their actions, that could disrupt an unprofessional act or model a professional one and even in taking steps after a professional lapse, raising a complaint or apologizing to patients. This study suggests that everyday resistance occurs frequently and takes a multitude of forms. Perhaps most interesting for our purposes, this study demonstrates the subtleties of everyday resistance in healthcare, highlighting acts that challenge or undermine professional lapses of more senior clinicians. Simple acts such as closing curtains for privacy when others have left them open or verbally challenging unprofessional behaviour were common. Many such acts of resistance went unnoticed (as resistance) by more senior staff.

Thinking about Australian immigration detention, we can begin to think of examples about how these systems or procedures may be undermined, how a nurse may omit information or emphasise certain points in emails or case notes. How rules may be ignored or how items may be smuggled into detention, just to name a few examples. When we look to the literature on Australian immigration detention and to the testimony of healthcare workers, we find few examples of such action. One exception comes from Dr Nick Martin, a General Practitioner who worked on Nauru:

The use of boat identification was ubiquitous when I started. Patients were referred to by all and sundry as ‘QLA027’ or similar and nobody batted an eyelid. I heard one of the IHMS bosses strenuously deny this practice at a Senate hearing, and laughed out loud at the lie. Later, once I had been in the job for a few months, I sat down with my team and explained that this was never to happen again: that these people were more than just numbers and we were to refrain from using numbers again. This went down surprisingly well, and word soon got around to new arrivals in the medical centre that this practice was now frowned on. For a few weeks people would begin quoting a boat number, stop suddenly, look slightly flustered and then recheck their piece of paper, and use the patients’ names (Martin 2018).

While initially confronting power, challenging members of his team, this example also shows how a simple shift in language from healthcare staff could begin to undermine one of the more dehumanising elements of immigration detention, that is, referring to detainees by number rather than name.

Healthcare professionals could also facilitate or turn a blind eye to other forms of everyday resistance within centres, those acts undertaken by detainees. On this point and looking elsewhere we can find further examples that begin to illustrate the forms that everyday resistance could take in this context. There is a growing literature that explores resistance as carried out by refugees and asylum seekers within border zones and immigration detention centres. Reporting on interviews with detainees formerly held in Australian immigration detention centres Bailey (2009) provides an example of the clandestine, everyday acts that were undertaken, as a means to undermine power:

Despite the harshness of the treatment they faced, detainees in Australian Immigration Detention Centres showed no respect for the Government’s attempts to contain them. On the decks of leaky boats they resisted attempts by the Australian Navy to turn them back. In serious danger at sea they engaged in hunger strikes and sabotage… They brought their politics across the threshold of the camp with them. Iranian trade union activists used their skills and experience to form committees and implement strike action. Iraqi leftists produced analyses of the camp and their prospects for freedom and African journalists translated them and turned them into bulletins. In the desert, behind barbed wire, under constant surveillance and subjected to brutal and unpredictable violence, their politics flourished. Secret networks planned escapes. Elections were conducted to facilitate representation. Mass meetings were held to decide action with translators relaying discussion across language and cultural barriers. As a result of this flourishing, their politics, their language and at times their bodies overwhelmed the fences and spilled into Australian cities. Bulletins were smuggled out and video cameras smuggled in. Mobile phones were thrown over fences. Phone link-ups were organised from outside to coordinate actions across the various detention centres. The centre pole of a Hills Hoist, an iconic Australian back-yard washing line, was used to lever apart bars (Bailey 2009).

Such resistance has unsurprisingly continued over the last decade. While detained on Manus Island, Behrooz Boochani wrote a book on a mobile phone using WhatsApp. It was smuggled out of Manus Island as thousands of PDF files. Since its publication Boochani’s book, No Friend but the Mountains (2018) has been widely praised and won a number of awards. While it has been labelled a number of things, this book also served as an act of resistance shining a light on offshore immigration detention on Manus Island, showing the dehumanising conditions, while at the same time maintaining a defiance toward these policies and their continuing colonising, racialized agendas (Boochani 2020). Boochani has continued writing since the publication of this book, however he was not the only one to resist while detained offshore. Sharples (2021) provides a more recent example, analysing 547 tweets from four accounts of those who were detained. This paper shows how such tweets have resisted the Australian governments discourse on offshore immigration detention. This was achieved in a number of ways, including normalising the presence of asylum seekers in the larger global phenomena of migration, humanising asylum seekers in the face of global discourses of dehumanisation, ensuring visibility by confirming the conditions of detention, highlighting Australia’s human rights violations and obligations, and challenging the government discourse on asylum seekers and offshore detention.

We find a number of other examples of resistance within immigration detention from across the globe. In a study conducted with 35 detainees in in a detention centre in Rotterdam, the Netherlands, van Houte et al. (2021) outlined a diversity of everyday resistance: from challenging ideas about belonging and citizenship to challenging the legitimacy of detention. The authors also outline acts of feigned or semi-compliance as acts of resistance. That is, detainees shared experiences and identified “bottlenecks” in deportation procedures, such as sharing the knowledge that “many embassies will not collaborate with return without valid proof of identity, especially when return is forced”. Those who employed such strategies did things such as destroy identity documents and made little effort to seek further identification. There were also those who engaged in more overt non-compliance, making it clear that they were concealing their identity and openly refusing to disclose their place of origin. Examples can also be found in Italy. In an ethnographic study with those in Lampedusa, Italy, Lendaro (2019) details how migrants refused to cooperate with authorities trying to determine their identities. In this case, migrants would refuse to have their fingerprints taken. As the authors note: “[h]aving one’s fingerprints taken would mean the start of a long and uncertain procedure which, in the best-case scenario, results in these migrants obtaining a status in a country in which they do not wish to settle”. The authors go on to outline how, this silent, individual protest snowballed, with more and more refusing to provide their fingerprints. This act, when collective “attain[ed] a subversive level of impact capable of sabotaging the system of classification and transfer of migrants”. This collective act also provided an opportunity for more overt forms of resistance, with migrants marching and occupying a public space, bringing this protest to the attention of the public and media. A final example come from the US and an ethnographic study of detention centres in New York. Instead of overt resistance, Kreichauf (2020) noted that most commonly resistance came in the form of “seemingly mundane and subtle everyday acts of disobedience”. This study outlines resistance through establishing and maintaining social relations and waiting, that is, while immigration detention forced detainees to wait, the fact that many continued to persevere in waiting provided them with some, however little, power.

## The advantages and justification for everyday resistance in detention

The value of everyday resistance might already be apparent to those who have either worked in detention or researched these issues. While healthcare professionals generally hold a relatively privileged position in society, those who work within detention are disempowered in a number of ways, unable to fulfill even the most basic of their patients needs; ultimately facilitating a harmful and unjust system. Even those who have spoken out publicly have done so with a great deal of risk; they have been frequently attacked or threatened by the Australian government. Everyday resistance provides a means to undermine and oppose this policy where overt dissent may be too risky. It also has several advantages to other concepts above. First, it politicises the act of providing care in detention. That is, framing an act as resistance implies it is political, not a clinical dilemma; it places the focus on the system as something that should be opposed. Such action is also consistent with the broader literature that rejects double standards for those who are detained (Farmer and Gastineau 2002). Second, it is consistent with efforts aimed at abolishing immigration detention and broader efforts of decarceration (Klonsky and Reinhart 2021). That is, such action complements broader efforts to undermine and oppose these policies and could work in coordination with these. For example, leaking information, whistleblowing or other coordinated subversive acts. On this point, and more practically steps should be taken to organise around such efforts, to facilitate and enable such action, and ultimately build networks of health workers who can support such efforts and build solidarity (Ganz 2010). Finally, we feel that resistance is a natural response to oppression, for health workers and detainees. On this point, we draw from Silvermint (2013) who argues that health and resistance are fundamentally related; resistance provides an opportunity not only to oppose oppression, but to also be involved in “valuable aims” or what is described as “goods, projects, relationships, and states of being that are important to the individual, as well as the general aim of leading a morally worthwhile life”.

While we have introduced a new strategy here, a form of action which could be adopted by healthcare workers in immigration detention and while we feel it has several advantages over other approaches, we still feel some of the other concepts above have merit and shouldn’t be dismissed completely. We feel in many ways, greater engagement with the literature on resistance could complement understandings about how to respond to dual loyalty conflicts or in responding to complicity with wrongdoing for example. It is also noteworthy, resistance isn’t and couldn’t be a supplement for all other clinical and ethical guidance. It should be seen amongst a set of potential actions that all may contribute to addressing the many issues as they relate to health and healthcare within immigration detention.

One further question that remains, related to the justification of such action. To our knowledge, resistance is not something that is discussed by any professional bodies nor can we find any guidance on such action. We feel that such action, broadly speaking, is consistent with the majority of guidance that already exists and other professional norms. Almost every major professional healthcare body in Australia has called for healthcare professionals within detention to uphold the dignity and rights of detainees. Everyday resistance provides a means of doing so when other means have failed. Furthermore, we believe that health workers, wherever possible we should resist injustice, particularly profound and completely avoidable injustices to which they contribute. For these reasons there appears to be a prima facie case for everyday resistance within immigration detention centres in Australia. In saying this however, everyday resistance is an umbrella term, in considering justification we also need to look at the potential impact and risks associated with each form of action. On the one hand everyday resistance could involve small subversive acts, such as pilfering or advocacy, on the other it could involve facilitating as escape attempt from detention. Each obviously has different ethical dimensions, potential impacts and risks. In beginning to think these issues through we are not without direction, we can draw on the broader literatures on resistance, such as civil disobedience, whistleblowing and strike action to begin to identify key features related to the justifiability of such action. In pursing everyday resistance a number of important questions could be asked. First, what are the aims of the action? Second, what are the chances of it being recognised by those in power and if it is recognised what are the potential repercussions? Third, are other actions available that could achieve the same outcome and that present fewer risks? Fourth, what are the trade-offs in failing to resist? While not exhaustive, each of these questions should be weighed carefully in considering whether everyday resistance can be justified. For example, action may be relatively high risk, but at the same time is unlikely to be recognised by those in power, on balance it may be the right thing to do. On the other hand, if such action is likely to be recognised, it may be preferable to employ another form of action or resistance. Finally, while above we have argued that everyday resistance is largely consistent with the statements made and guidance available from all major health bodies in Australian, we do not speak for them, nor can we completely assume their response to such action. Beyond the Australian government, healthcare professionals should also consider the risks to themselves and the profession. There is no clear ‘official’ guidance of such action, nor how healthcare professionals should undermine or oppose injustice more broadly. The regulatory questions that such action raises needs greater discussion and engagement from professional bodies, their involvement in outlining a minimum standard for healthcare within detention has already been called for (Dudley et al. 2020).

## Conclusions

Everyday resistance is a strategy that is often employed by groups who have little power and luxury for open resistance. It includes acts such as feigned ignorance, non-compliance and sabotage and can be compared to more open, public forms of action such as civil disobedience, marches and sit-ins. Everyday resistance needn’t be identified by those in power so often comes with far fewer risks than open opposition.

While few example exist from Australian immigration detention (perhaps unsurprisingly because of the very nature of the action), we have provided a number of examples of different forms of everyday resistance within immigration detention from across the globe. We have argued that such action has the potential to provide a means to undermine injustice, a means to restore the rights of those detained and a means for healthcare professionals to act in their patients best interests. We have also argued that there is a prima facie justification for such action, and offered some suggestions in examining the justifiability of specific acts of resistance. There is of course much more that could be said about everyday resistance, it remains a conceptually disputed topic in itself, its relationship with power is also debated. Then there are of course the range of normative questions beyond the brief discussion above. Beyond what I have discussed here, the related issue also remains about how clinicians should position themselves in response to more overt forms of opposition, such as protests within detention (Fiske 2016). What for example is the relationship for detainees between their distress, their mental disorder/illness and their sometime incapacities (as diagnosed by health professionals and narrated by themselves), and their resistance (Fiske 2012, p183) It should go without saying that there is fertile ground for greater discussion on these points.

In offering a defence of resistance, Ferrell (2019) asserts that “if we don’t have the ability to kick open the door to a better world, we’d best learn how to pick the lock”. While we should continue to oppose Australian immigration detention and take action to demand change, while we are waiting and where there is not the luxury for open resistance, we can and should undermine, sabotage and more generally resistant wherever we can. Healthcare workers within detention centres, while extremely limited in what they can do, continue to have the power to resist. Such action has the potential to undermine injustice and uphold the dignity of those detained.

## References

[CR1] Amnesty International. 2016. “Island of Despair: Australia’s “processing” of refugees on Nauru”.

[CR2] Australian Human Rights Commission. 2014. *National inquiry into children in immigration detention (Forgotten Children). Transcripts from Third Public hearing, 31 July 2014*. “.

[CR3] Baaz, M., M. Lilja, M. Schulz, and S. Vinthagen. 2016. Defining and analyzing resistance possible entrances to the study of subversive Practices, *Alternatives*, Vol. 41 No. 3, pp. 137–153.

[CR4] Bailey R (2009). Up against the Wall: Bare Life and Resistance in Australian Immigration Detention. Law and Critique.

[CR5] Boochani, B. 2018. *No friend but the mountains: writing from Manus Prison*. Picador Australia.

[CR6] Boochani, B. 2020. “Opinion:‘White Australia’Policy Lives On in Immigrant Detention”, *The New York Times*.

[CR7] Briskman L (2020). The people’s inquiry into detention: social work activism for asylum seeker rights. Journal of Sociology.

[CR8] Briskman L, Zion D (2014). Dual loyalties and impossible dilemmas: Health care in Immigration Detention. Public Health Ethics.

[CR9] Doherty, B. 2020. “Australia’s offshore detention is unlawful, says international criminal court prosecutor”, *The Guardian*.

[CR10] Doherty, B., and H. Davidson. 2016. *Save the children workers unfairly fired on Nauru for political reasons – report*. The Guardian Australia.

[CR11] Dudley M (2003). Contradictory australian National policies on self-harm and suicide: the case of Asylum Seekers in Mandatory Detention. Australasian Psychiatry.

[CR12] Dudley, M., P. Young, L. Newman, F. Gale, and R. Stoddart. 2020. “Health professionals confront the intentional harms of indefinite immigration detention: an Australian overview, evaluation of alternative responses and proposed strategy”, *International Journal of Migration, Health and Social Care*.

[CR13] Ellefsen R, Banafsheh A, Sandberg S (2022). Resisting racism in everyday life: from ignoring to confrontation and protest. Ethnic and Racial Studies.

[CR14] Essed, P. 1991. *Understanding everyday racism: An interdisciplinary theory*, Sage.

[CR15] Essex, R. 2016a. A Community Standard: Equivalency of Healthcare in Australian Immigration Detention, *Journal of Immigrant and Minority Health*, pp. 1–8. 10.1007/s10903-016-0438-7 27220966

[CR16] Essex, R. 2016b. Healthcare and Complicity in Australian Immigration Detention, *Monash Bioethics Review*10.1007/s40592-016-0066-y 27757831

[CR17] Essex, R. 2018. Should clinicians boycott australian immigration detention?, *Journal of Medical Ethics*, pp. medethics-2018-105153. 10.1136/medethics-2018-105153 30463934

[CR18] Essex R (2019). Do codes of ethics and position statements help guide ethical decision making in australian immigration detention centres?. BMC Medical Ethics.

[CR19] Essex, R. 2020. *The Healthcare Community and Australian Immigration Detention: the case for non-violent resistance*. Palgrave MacMillan.

[CR20] Essex, R. 2021. “Resistance in health and healthcare”, *Bioethics*. 10.1111/bioe.12862 33683714

[CR21] Farmer P, Gastineau N (2002). Rethinking health and human rights: time for a paradigm shift. Journal of Law Medicine & Ethics.

[CR22] Ferrell, J. 2019. In defense of resistance, *Critical Criminology*, pp. 1–17.

[CR23] Fiske, L. 2012. *Insider resistance: understanding refugee protest against immigration detention in Australia, 1999–2005*. Curtin University.

[CR24] Fiske, L. 2016. *Human Rights*. Refugee Protest and Immigration Detention”.

[CR25] Ganz, M. 2010. “Leading Change: Leadership, Organization, and Social Movements.“ Edited by Nitin Nohria and Rakesh Khurana. Handbook of Leadership Theory and Practice.

[CR26] Garton, J. 2017. “Topic: Neo-colonialism and Australian off-shore detention”.

[CR27] Green JP, Eagar K (2010). The health of people in australian immigration detention centres. Medical Journal of Australia.

[CR28] Hayward K, Schuilenburg M (2014). To resist = to create? Some thoughts on the concept of resistance in cultural criminology. Tijdschrift over Cultuur & Criminaliteit.

[CR29] Jansen, M., A. S. Tin, and D. Isaacs. 2018. Prolonged immigration detention, complicity and boycotts, *Journal of Medical Ethics*, pp. medethics-2016-104125. 10.1136/medethics-2016-104125 28794237

[CR30] Klonsky, A., and E. Reinhart. 2021. As Covid Surges Again, Decarceration Is More Necessary Than Ever. *The Nation*. https://www.thenation.com/article/society/covid-prisons-decarceration/.

[CR31] Koutroulis G (2009). Public health metaphors in australian policy on asylum seekers. Australian and New Zealand Journal of Public Health.

[CR32] Koziol, M. 2018. Australia’s chief medical officer on Nauru deported amid health policy crisis, *The Sydney Morning Herald*

[CR33] Kreichauf, R. 2020. Detention as Social Space: Waiting, Social Relations, and Mundane Resistance of Asylum Seekers in Detention, *Critical Sociology*, p. 0896920520977644.

[CR34] Lendaro, A. 2019. Nothing to lose: the power of subtle forms of resistance in an immigration detention centre. In *Governance beyond the Law*, 309–322. Springer.

[CR35] Lepora, C., and R. E. Goodin. 2013. *On complicity and compromise*. Oxford University Press.

[CR36] Lilja, M. 2022. The definition of resistance, *Journal of Political Power*, pp. 1–19.

[CR37] Litz BT, Stein N, Delaney E, Lebowitz L, Nash WP, Silva C, Maguen S (2009). Moral injury and moral repair in war veterans: a preliminary model and intervention strategy. Clinical Psychology Review.

[CR38] Mainey, L., C. O’Mullan, and K. Reid-Searl. 2022. Working with or against the system: Nurses’ and midwives’ process of providing abortion care in the context of gender-based violence in Australia, *Journal of Advanced Nursing*10.1111/jan.15226 35285985

[CR39] Martin, N. 2018. “The Nauru Diaries”, *Meanjin*.

[CR40] Physicians for Human Rights. 2002. “Dual loyalty and human rights in health professional practice; Proposed guidelines & institutional mechanisms”, *Retrieved from* http://physiciansforhumanrights.org/library/reports/dual-loyalty-and-human-rights-2003.html

[CR41] Reeders, D., and S. Nguyen. 2014. Defining and responding to everyday racism, *Croakey Health Media*

[CR42] Sanggaran, J.-P. 2016. *First, do no harm. Why doctors should boycott working in australian detention centres*. The Guardian Australia.

[CR43] Scott JC (1986). Everyday forms of peasant resistance. The Journal of Peasant Studies.

[CR44] Scott JC (1989). Everyday forms of resistance. The Copenhagen Journal of Asian Studies.

[CR45] Scott, J. C. 1990. *Domination and the arts of resistance: hidden transcripts*. Yale university press.

[CR46] Sharples R (2021). Disrupting State Spaces: Asylum Seekers in Australia’s Offshore Detention Centres. Social Sciences.

[CR47] Shaw MK, Rees CE, Andersen NB, Black LF, Monrouxe LV (2018). Professionalism lapses and hierarchies: a qualitative analysis of medical students’ narrated acts of resistance. Social Science & Medicine.

[CR48] Shutzberg M (2019). Unsanctioned techniques for having sickness certificates accepted: a qualitative exploration and description of the strategies used by swedish general practitioners. Scandinavian Journal of Primary Health Care.

[CR49] Shutzberg, M. 2021. *Tricks of the Medical Trade: Cunning in the age of bureaucratic austerity*. Södertörns högskola.

[CR50] Silvermint D (2013). Resistance and well-being. Journal of Political Philosophy.

[CR51] van Houte, M., A. Leerkes, A. Slipper, and L. Breuls. 2021. Globalised citizenship and the perceived legitimacy of immigration control: narratives and acts of resistance in immigration detention, *Migration Studies*

[CR52] Vinthagen S, Johansson A (2013). Everyday resistance: exploration of a concept and its theories. Resistance Studies Magazine.

[CR53] Zion D, Briskman L, Loff B (2012). Psychiatric Ethics and a politics of Compassion. Journal of Bioethical Inquiry.

